# ‘Hampers’ as an effective strategy to shift towards sustainable diets in South African low-income communities

**DOI:** 10.1080/0376835X.2022.2028605

**Published:** 2022-01-27

**Authors:** Madeleine Coste, Laura Pereira, Andrew Charman, Leif Petersen, Corinna Hawkes

**Affiliations:** a Centre for Food Policy, City University of London, London, UK; b Stockholm Resilience Centre, Stockholm University, Stockholm, Sweden; c Global Change Institute, University of the Witwatersrand, Johannesburg, South Africa; d Sustainable Livelihoods Foundation, Cape Town, South Africa

**Keywords:** Hampers, food security, sustainable food systems, Cape Town, food retail

## Abstract

Transitioning towards sustainable diets is imperative to avoid the worst effects of climate change, environmental degradation, and malnutrition. In South Africa, households most vulnerable to food insecurity employ various strategies to access food. These include purchasing hampers; a combination of staple foods sold in bulk at a discounted price, which are cake wheat flour, super maize meal, white sugar, cooking oil, and white parboiled rice. We explore the barriers and opportunities for hampers to advance sustainable diets in the context of Cape Town. Our findings show hampers contain energy-dense, nutrient-poor foods. Furthermore, we find that brand loyalty plays an important role in households’ purchase of hampers. We conclude there is potential to leverage hampers to become a sustainable strategy through which people can access healthier food by working with retailers to offer nutritious and sustainably produced alternatives. Such change would require challenging retailers’ and consumers’ understanding of what ‘necessities’ are.

## 1 Introduction

The current global food system is not meeting the food needs of the planet’s citizens whilst it continues to erode the biodiversity and ecosystems upon which it depends (IPBES, 2019). Transitioning towards sustainable diets is therefore imperative to avoid the worst effects of climate change, environmental degradation, and malnutrition- and to ensure long-term food security for all the planet’s people. Countries that enable their populations’ access to a diet that is both healthy and sustainable are expected to benefit from drastic reductions in health care costs and costs associated with environmental degradation (Willett et al., 2019). However, this can only be achieved through developing sustainable food systems, involving all actors and activities in the production, processing, distribution, preparation, consumption, and disposal of food (FAO, 2014).

Sustainable food systems are especially critical to promote in emerging countries that are likely to experience both the worst consequences of climate change and the burden of an ongoing shift towards unhealthy diets, high in calories and heavily processed and animal source foods (Willett et al., 2019). These impacts are exacerbated by rapid urbanisation, increasing disposable income for food, and the persistent issue of inadequate access to nutritious foods (Willett et al., 2019). Emerging economies, such as South Africa, are increasingly facing multiple burdens of malnutrition, where society faces high rates of undernutrition from a lack of calories, micronutrient deficiencies, and high rates of overweight and obesity from an excess of calories (Development Initiatives, 2018). Although South Africa is food secure at the national level, more than 20% of households have inadequate or severe inadequate access to meet their food needs (StatsSA, 2017). Food insecurity is also becoming an increasingly urban problem where over 60% of households that experience hunger live in urban areas (Battersby, 2017; StatsSA, 2018). As a result of widespread food insecurity, in both urban and rural areas, households are forced to find strategies to help stretch incomes as far as possible when procuring food. However, the long-term resilience of such strategies in promoting more sustainable diets – that ensure not just health outcomes, but also environmental sustainability – are not well known.

One key strategy of low-income households in South Africa is to purchase ‘hampers’. According to Bowden et al. (2018), a hamper can be defined as a bundle of goods considered staples, including maize meal, flour, oil, rice and sugar, bought at a discount price from either formal or informal stores, as a strategy to access food cheaply. In her book, Joubert (2012:176) describes hampers by saying: ‘the benefits for people with such meager resources is that they get all the basics they will need for a month, without having to trawl the shelves of their local shop and decide how to divvy up the monthly budget’. Although bulk-buying is a common strategy among low-income urban households, the concept of hampers as they are promoted in South Africa remains unexplored in academic research (Bowden et al., 2018).

In this paper, we address this gap by describing hampers as a current strategy to combat hunger within the South African food system. Through a case study in Cape Town, we describe what hampers typically contain, how and why they are purchased, their supply chain and connection to ‘Big Food’, and their nutritional content. We conclude with a discussion of hampers as a long-term food security strategy and the implications of these findings for the region.

### 1.1 An overview of the South African food system

The ‘tendency of poor people to lean on cheap, high-energy, low-nutrient foods is driving an apparently contradictory situation where you increasingly find a malnourished individual living inside the skin of a fat or obese person’ (Joubert, 2012:9). Like other emerging countries, South Africa is seeing rapid changes in its population’s diet and the emergence of new diet-related illnesses alongside persistent food insecurity. Although South Africa produces enough calories to feed its population, food insecurity remains an important concern (Drimie & McLachlan, 2013): according to the latest South African National Health and Nutrition Examination Survey (SANHANES-1), hunger is prevalent in 26% of the population, 30.3% of the black population is food insecure, and stunting is the most common nutritional disorder, affecting 26.5% of children (Shisana et al., 2014). A 2005 national survey of children aged 1–9 years old showed that 9.3% of children surveyed were underweight (Stupar et al., 2012).

South Africans are meanwhile quickly adopting a ‘Westernised diet’, characterised by an increased consumption of energy-dense processed and industrial foods containing high quantities of sugar, salt, and fat, and are shifting away from unrefined grains and starchy roots, legumes, vegetables and fruits (Stupar et al., 2012). In comparing per capita consumption of various food products between 1994 and 2012 in South Africa, a decline is observed in the consumption of traditional crops (such as millet, sorghum) and an increase in the consumption of rice by 48% between 1999–2012, of vegetable oils by approximately 30%, of total sugar consumption by 33% (included in sugary drinks and processed foods) and an increase of breakfast cereals consumption of around 43% (Ronquest-Ross et al., 2014). This nutrition transition has resulted in an increase in national overweight and obesity rates, which reached 50% for women, and 30% for men in 2012 (Shisana et al., 2014), as well as an increase in non-communicable diseases (NCDs) such as cardiovascular disease (CVD), diabetes, and diet-related cancers, particularly in urban areas (Muzigaba et al., 2016; Thow et al., 2016).

Whilst many factors may explain South Africans’ shift to Westernised diets, some key trends must be highlighted. Firstly, de-agrarianisation and urbanisation trends have increased dependence on purchasing of food, which in turn has meant an increased reliance on social grants to meet food needs (Pereira, 2014). Further, Temple et al. (2009) find that healthy foods may cost between 30% and 110% more when compared to less healthy alternatives based on the cost of food energy. For many, food remains expensive despite government efforts to make food more affordable to poorer households. South Africa’s Value-Added Tax (VAT) system that was introduced in 1991 exempts a number of basic foodstuffs of VAT, as a way to provide relief to low-income households who spent a relatively high proportion of their income on these items. This list includes 19 items, among which feature rice, cooking oil, brown bread, maize meal, milk, eggs, certain fruits, and vegetables (National Treasury of South Africa, 2018). On April 1, 2018, the general VAT rate increased from 14 to 15%. One year later, the list of VAT-free food items was expanded to include two more food items, cake wheat flour and white bread flour, by recommendation of an independent panel of experts, in a study commissioned by the Ministry of Finance. This recommendation ‘was based on the increased consumption of cake flour and the uses thereof by the poor’ (National Treasury of South Africa, 2018:48).

Secondly, the rise of supermarkets is also complicit in this dietary shift. Supermarkets represent about 2% of the number of food retail outlets by number but have an estimated retail share of 50–60% (McLachlan & Landman, 2013). On the margins of this formal commercial sector operates an informal economy that constitutes a very high number of small-scale farmers, traders, and small informal shops called ‘spazas’ (Pereira & Drimie, 2016; Charman et al., 2019; Petersen et al., 2019). Supermarket expansion has detrimentally impacted food access for the poor as they are often incompatible with the consumption strategies of the poorest households, most notably their reliance on informal vendors for fresh produce (Peyton et al., 2015). Supermarketisation has also resulted in an increased availability of and access to the products of ‘Big food’ that are implicated in the nutrition transition (Igumbor et al., 2012).

### 1.2 The case study: Cape Town, South Africa

Cape Town is a coastal city of 3.74 million people (StatsSA, 2011), which includes the city centre and the ‘Cape Flats’ to the east where most townships and informal settlements are located. According to the latest census, over 35% of Cape Town households live below the poverty line of 3500 South African rand (ZAR) a month (StatsSA, 2011). Cape Town also experiences high rates of urban food insecurity; according to a survey of 1060 households in three of the city’s low-income areas (Ocean View, Philippi and Khayelitsha), 80% of households were moderately or severely food insecure and only 10% of households in Khayelitsha and Philippi were classified as ‘food secure’ (Battersby, 2011). For low-income communities in Cape Town, food was found to represent over half of total house-hold expenditures (Frayne et al., 2010).

## 2 Methods

Very little literature is available on hampers; therefore an exploratory approach was taken to extend on the findings of Bowden et al. (2018), Battersby (2011) and Joubert (2012), which make references to hampers and bulk-buying in South Africa. The research was conducted through a mixed-methods approach, including a literature review, collection of store-level data, qualitative interviews and thematic analysis, quantitative analysis of survey data collected by the Sustainable Livelihoods Foundation (SLF) in Philippi (SLF, 2018; Charman et al., 2019), and a mapping of the supply chain actors, informed by the literature review and interviews (Table 1). Finally, the nutritional and environmental footprints of the hamper contents were calculated using information either directly on the goods’ packets or from the literature.

### 2.1 Data collection

**Figure 1:**
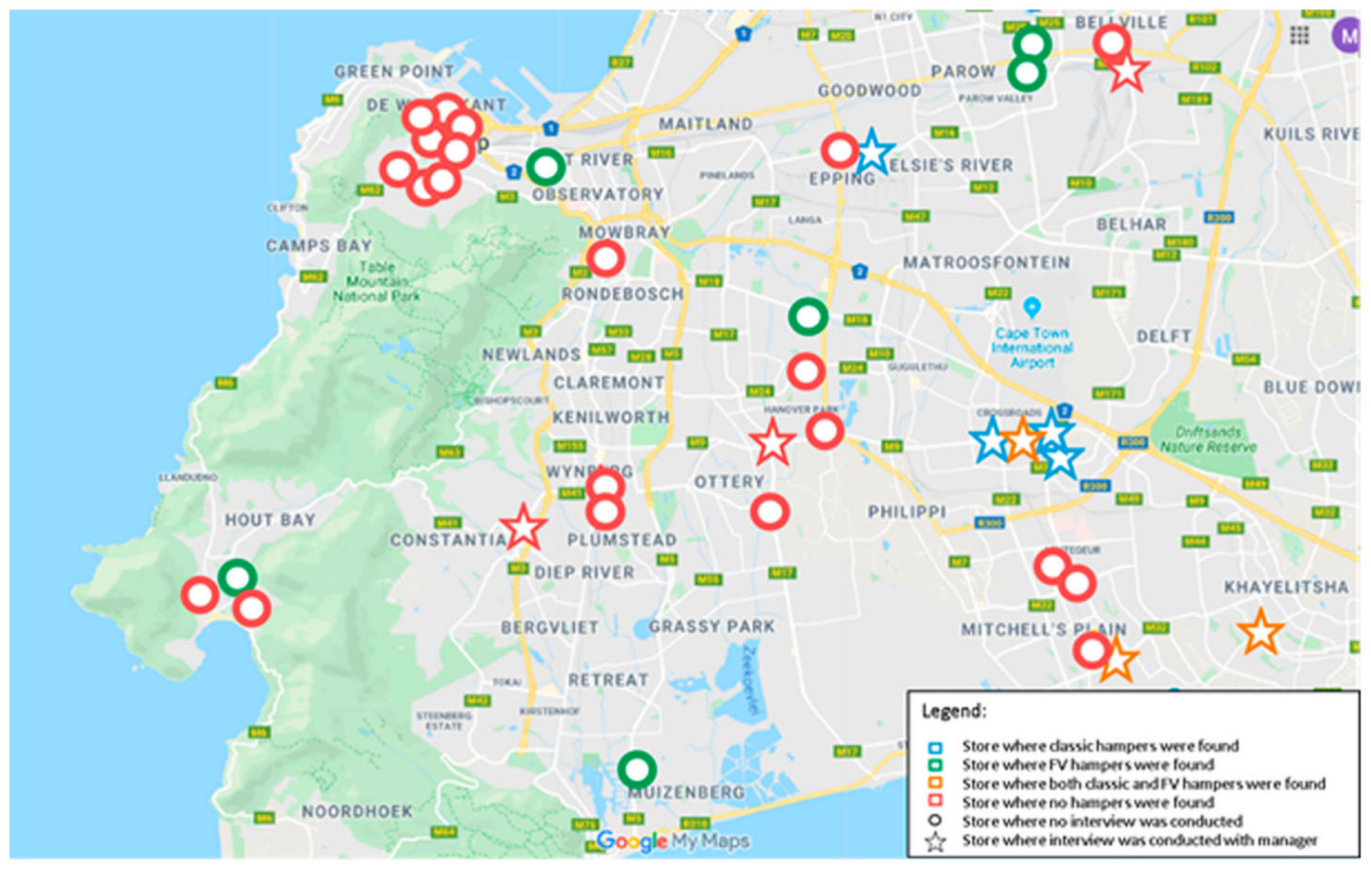
Map of formal stores visited during fieldwork in Cape Town.

The first step of this study was to identify where hampers can be found across Cape Town. In addition to the ‘classic’ hamper, fruit and vegetable hampers sold at a discounted price can also be found. The map below (Figure 1) shows 37 formal stores visited during the field work in July 2018 located in the City Centre, Northern Suburbs (Parow, Bellville), Cape Flats (Epping, Khayelitsha, Philippi, Ottery, Mitchell’s Plain), Southern Suburbs (Muizenberg, Wynberg) and Hout Bay. These were selected based on location to ensure that data was collected from a range of suburbs of varying income. Figure 1 shows that classic hampers were found in 7 stores visited, in supermarkets and wholesalers, all of which located in the region of the Cape Flats (Epping, Khayelitsha, Philippi, Ottery, Mitchell’s Plain). Fruit and vegetable hampers were found in 9 stores. From the informal shops visited, 3 hampers were found, indicating that hampers can be purchased in some spaza shops.

Fieldwork was conducted in July 2018 in Cape Town with primary data was collected in 37 formal stores located in different parts of the city (Figure 1). The selection of formal stores included each of the national grocery retail sector’s major players according to market share (Table 2). To understand how the formal and informal markets differed, 3 spazas in the City Bowl and Wynberg were also visited. The data collected included:

(1) Information about where hampers were sold, which specific products and brands they contained, and their price.
(2) Information about a range of food products including bread, canned pilchards, dry beans, maize, flour, sugar, oil, and rice, initially thought may be included in hampers before the precise content of hampers was narrowed down to the 5 staples. For over 900 products, information relating to the type, price, brand, shelf display and promotions, nutritional content (nutritional label), country of origin and name of the processing company of the products was collected.

Semi-structured qualitative interviews were conducted in English with managers from 10 formal retail stores detailed in Table 2. Seven interviews were conducted in stores which sold hampers, 2 in stores located outside the Cape Flats which didn’t sell hampers, and 1 in Philippi where no hampers were found. The aim of the interviews was to understand from store managers the origin and content of hampers, who purchases them, how they are priced, who decides the contents of hampers and whether this varies. Interviews lasted between 10 and 30 min and were audio-recorded.

The fieldwork data cannot be considered as representative of general trends; the data collected is time- and space-specific, prices may vary weekly and monthly. Secondly, the sources of data cover different geographical scopes, but offer insights into broader trends to be further explored.

### 2.2 Consumer survey data

To triangulate the data collected from the interviews, primary data from the SLF was used to provide an in-depth picture of hampers in the Cape Flats. In June 2018, the SLF conducted research in the Philippi township to understand households’ food purchasing habits from the Phola Park and Better Life settlements. The dataset included survey responses to over 70 questions from 118 heads of households, relating to their food purchasing habits from formal and informal stores (SLF, 2018; Charman et al., 2019).

### 2.3 Analysis

Store-level data was compiled into Excel and analysed to identify patterns including the number and location of stores which sold hampers, the products and brands most frequently found in hampers, the pricing of hampers, and how these results compare with the same products sold individually on the shelves. Interviews were transcribed in full into Excel, and thematic analysis was used to identify themes and patterns (Riessman, 2005). Interview responses were coded using *invivo* coding, in which codes were based on language used by interviewees (Saldaña, 2014). Codes were organised into subthemes under 5 overarching themes; (1) Description of customers, (2) Hamper pricing, (3) Hamper contents and brand loyalty, (4) Business decisions and food industry actors, (5) Hamper origins.

Data collected by the SLF was used to complement the analysis with insights on low-income households’ consumption habits of hampers. The themes explored included (1) where households shopped, (2) factors influencing shopping decisions, (3) preferred brands, (4) frequency and location of shopping, (5) purchase of hampers and how.

The analysis was conducted to find patterns in the data, by looking at average responses and correlations between variables.

### 2.4 Nutritional information and actor mapping

Store-level data was used to evaluate the nutritional content of hampers. Once the specific products and brands most frequently found in hampers were identified, these products’ packaging labels detailing their nutritional quality, geographical origin and food processing company that manufactured them were used for the analysis. Additional desk-based research further helped understand the wider implications hampers have on the food system at the national level.

The EAT-Lancet Commission uses a ‘reference diet’ considered a healthy diet providing an adult with all necessary nutrients (Willett et al., 2019). Thus, a healthy diet should include a variety of foods from different food groups, including fruit and vegetables, proteins, fat, carbohydrates, and little to no sugar. An analysis was made to compare the macro- and micronutrient content of a sample hamper found in a store in Philippi, with the EAT-Lancet Commission’s reference diet.

## 3 Results

### 3.1 What are hampers?

**Figure 2:**
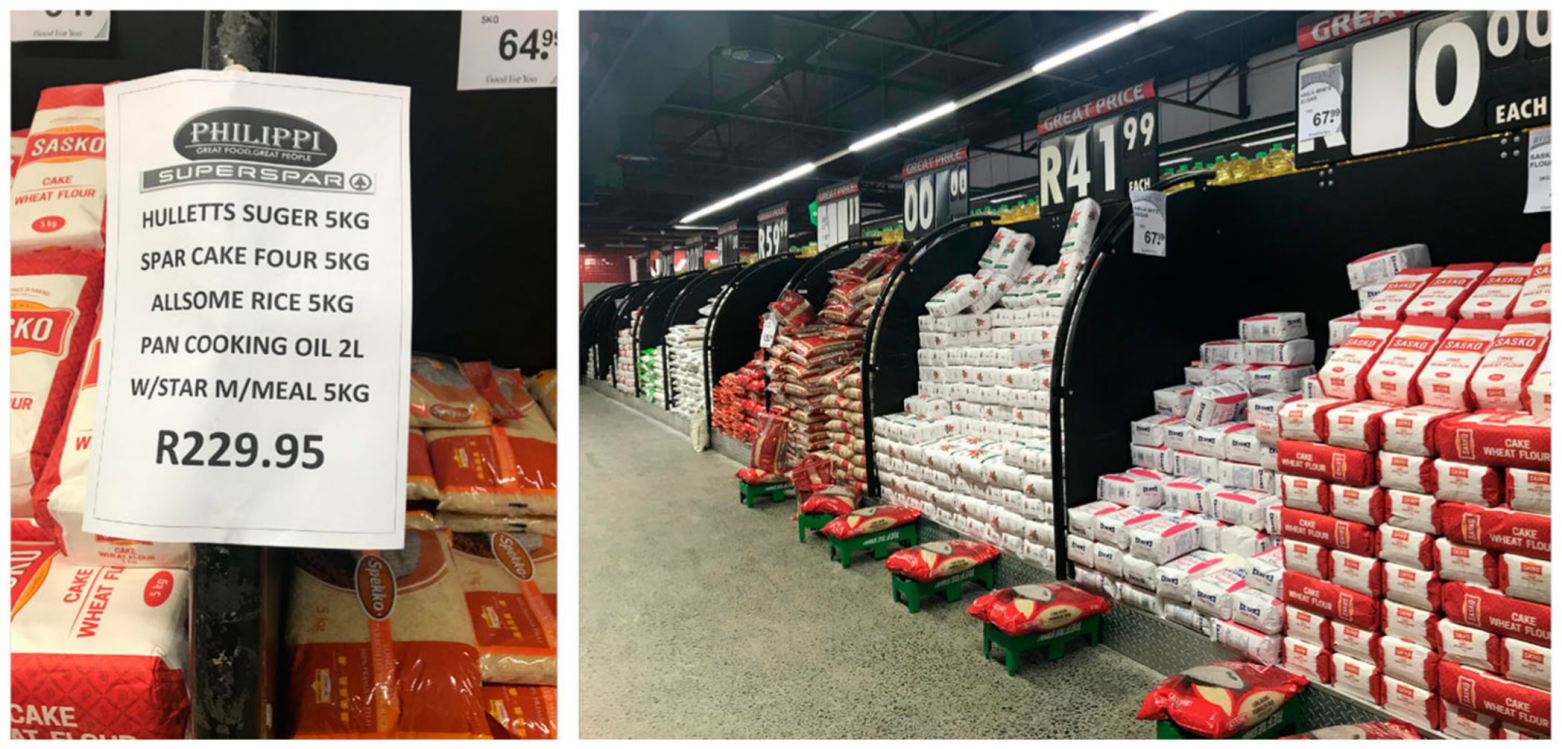
Pictures of a classic hamper in Philippi Superspar (Source: Author’s own).

Fourteen types of hampers were found across 7 of the 37 formal stores visited across Cape Town, in both supermarkets and wholesalers, all of which were situated in the Cape Flats. Two more hampers were found in spazas in Wynberg, although they presented significant differences with the ‘classic’ hamper. Hampers, often referred to as ‘combos’, typically constituted a combination of 5 staple foods sold at a discounted price, in bulk: these included cake wheat flour, super maize meal, white sugar, cooking oil, and white parboiled rice. Typically, each product was sold in packages of 5 or 10 kgs, and in bottles of 2 l for cooking oil. The hampers found in formal stores were not packaged together; in most cases, items were shelved individually, placed in a way to catch the customers’ eye with a sign nearby indicating the items and brands forming the hamper and its price, as seen in Figure 2. Several shops offered multiple hampers, composed of the same 5 products but from different brands.

**Figure 3:**
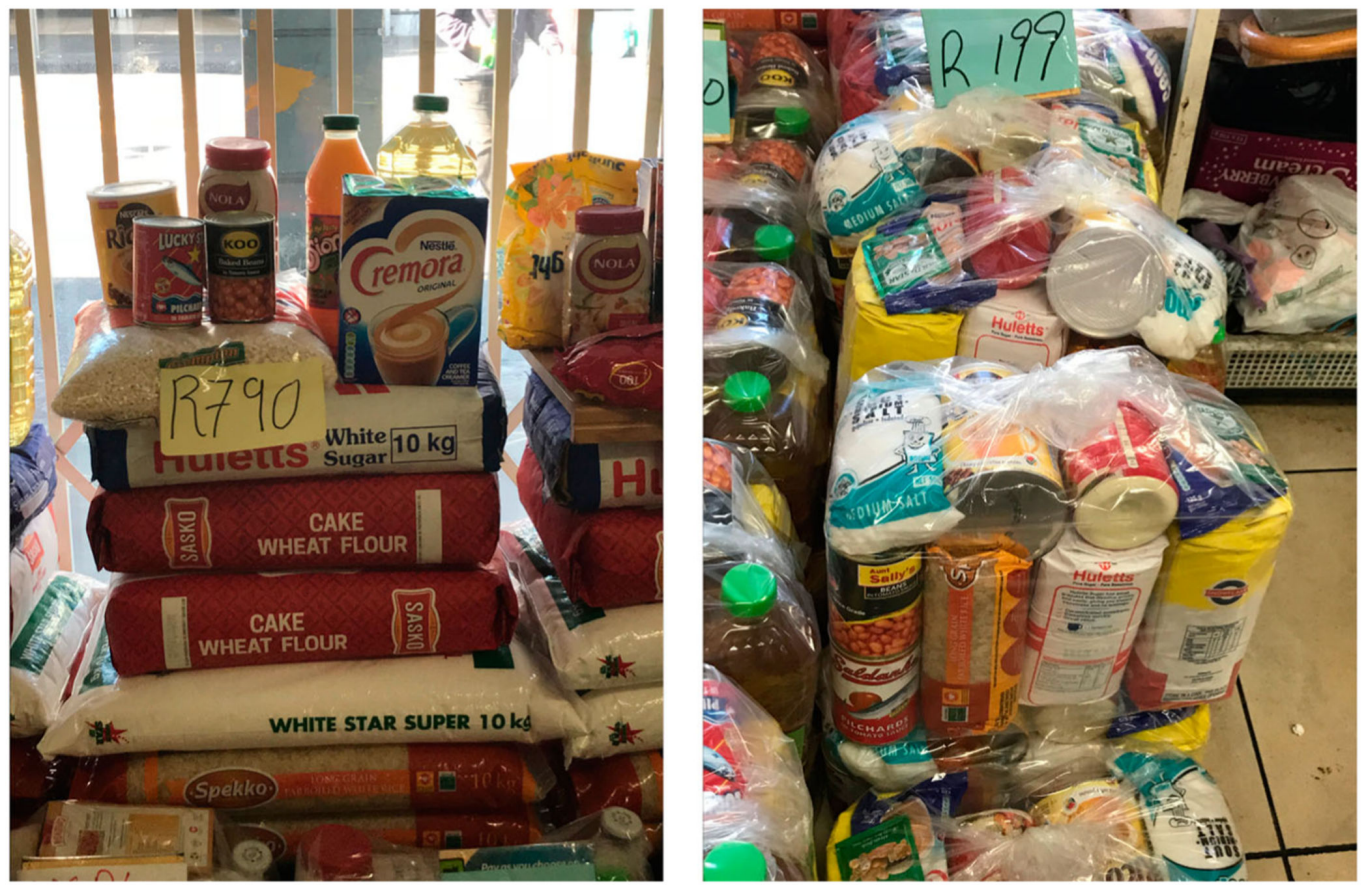
Spaza hampers (Source: Author’s own).

As Figure 3 shows, hampers found in spazas are generally packaged in a bag for more convenient pickup and transportation, and include a wider variety of products, on top of the 5 staples, including canned beans, pilchards, mayonnaise, and coffee cream for example.

**Figure 4:**
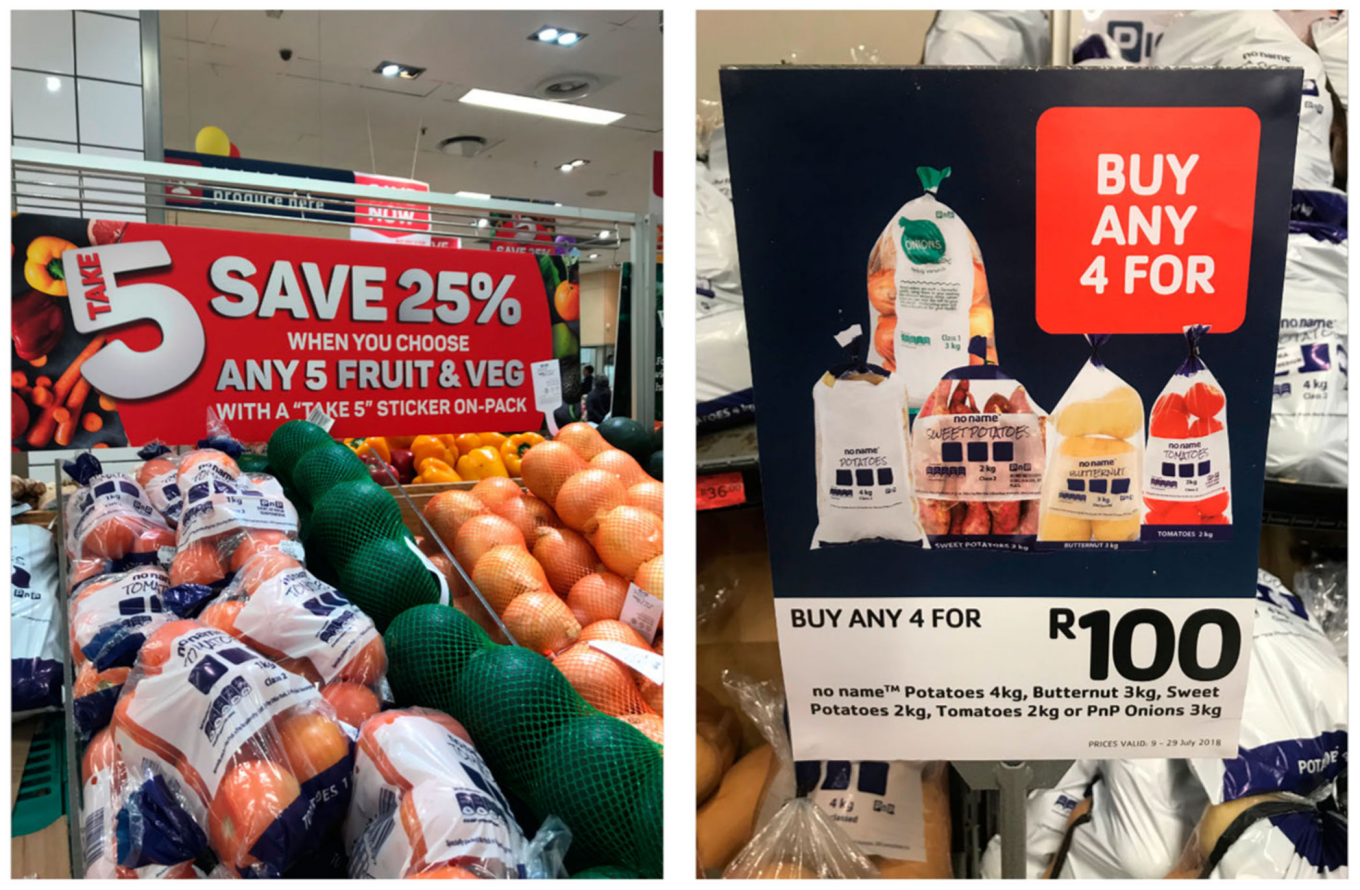
Pictures of FV hampers at Pick n Pay (Source: Author’s own).

Nine stores sold fruit and vegetable hampers (featured in Figure 4) and were widely distributed across the Cape Town municipality, including the City Bowl, Atlantic Seaboard (Hout Bay), Northern Suburbs (Parow, Bellville), South Peninsula (Capricorn) and Cape Flats (Khayelitsha, Gatesville, Philippi). SLF found that fruit and vegetable hampers from Goal and Spar usually contained potatoes, carrots, onions, and butternut (Charman et al., 2019).

### 3.2 Where and why are hampers sold?

Hampers were found in several Shoprites, Spars, as well as Boxer and Goal food retail stores. The research shows that hampers are also sold in the informal sector; corroborated by a project conducted by SLF on spazas in March 2017 which registered 19 shops selling hampers (SLF, 2018). In the SLF household survey, 89 of the 118 respondents interviewed in Philippi (74%) said they purchase hampers from supermarkets; 63% of whom purchase hampers monthly (Charman et al., 2019). Goal was the most popular store for purchasing food, and hampers were one of the two main reasons why they shopped there, along with the perception that food was sold at a ‘lower price than at their competitors’.

\Store managers are targeting low-income households with hampers; interviewees responded that the idea of a hamper was to help customers ‘stretch the rand’ (**SM1**, **SM4**). Six managers explained that the decision to sell hampers and what they are composed of, lies with the individual store managers, rather than with headquarters. Store managers described a similar method to pricing their hampers in order to remain competitive; they assemble hampers by selecting a combination of staples that headquarters had decided to put on promotion, and of brands preferred by customers, and next determine a discount price with the aim of making the hamper as cheap as possible. Because hampers are very similar in nature and include broadly the same products and brands, stores use low-priced hampers as ‘draw cards’ to attract customers (**SM1**, **SM4**).

Price data was collected on both the hampers and on individual store items to estimate the savings made on the purchase of hampers. Table 3 shows that 4 of the 14 hampers seemed to fail to generate any savings for the customer (cells in red). Four hampers were estimated to generate less than 5% of savings. Six were estimated to provide savings ranging from 5 to 18%. Analysis by the SLF found similar results for spazas where hampers provided less 1.5% of savings compared to buying items separately (Charman et al., 2019).

**Figure 5:**
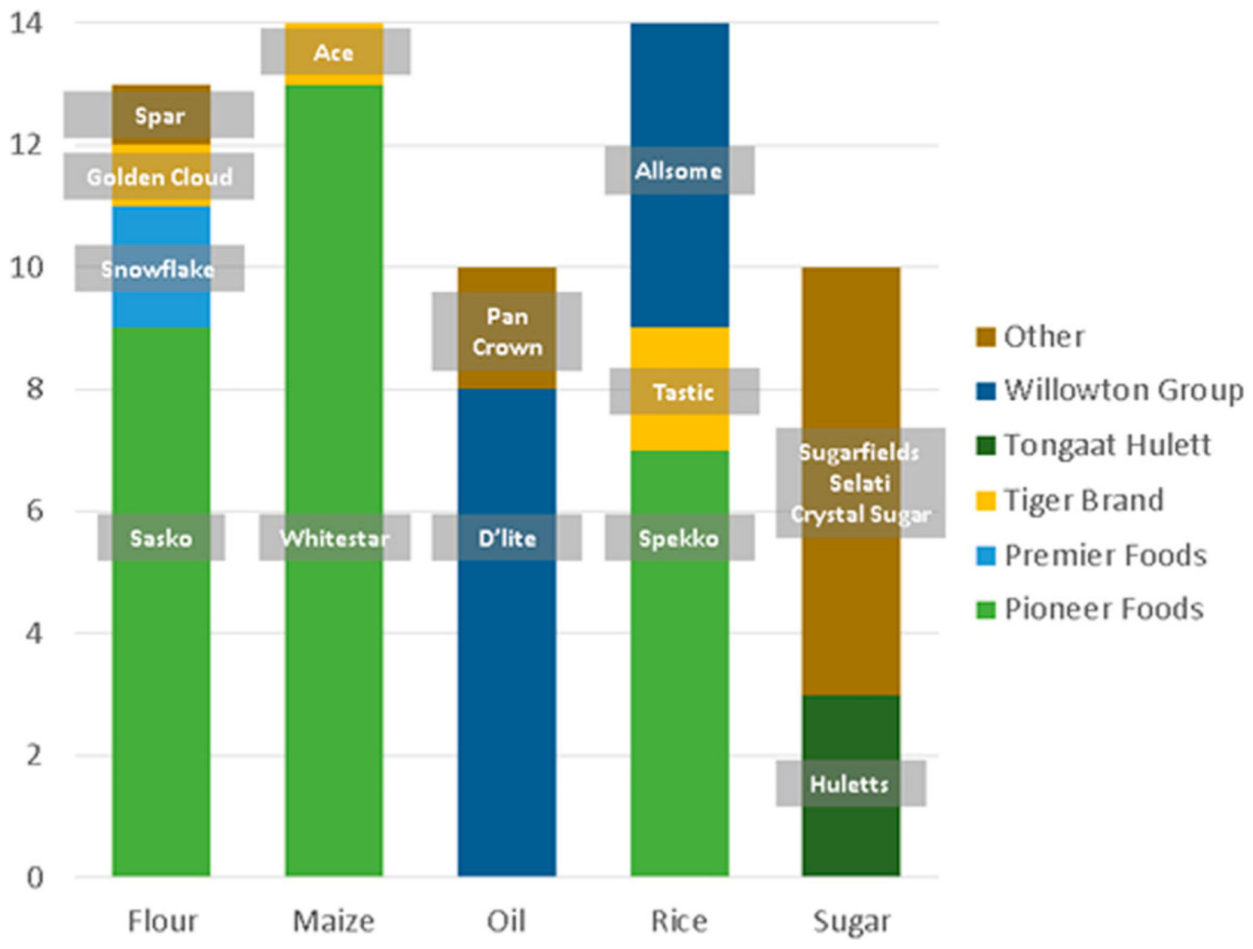
Main products and brands comprising hampers, and the processing companies that produce them.

In addition to pricing, the brand of individual products included in hampers was identified as an important factor in consumers’ purchasing decision. Three interviewees talked about the importance of ‘brand loyalty’ (**SM1**, **SM5**, **WM2**) and responded that customers purchased their hampers because they contained brands that they ‘liked’ or ‘loved’ (**SM1**). Particularly, *Whitestar* maize meal and *Spekko* rice (both belonging to Pioneer Foods) emerged as very popular brands and were most frequently included in hampers. Figure 5 shows how often each product and brand was found in the 14 hampers from the formal sector, as well as the processing companies the brands belong to. The most popular products were *Whitestar* and *Iwisa* maize meal, *Sasko* and *Snowflake* cake wheat flour, *Spekko* and *Allsome Rice* parboiled white rice, *Crystal* and *Huletts* white sugar, and *D*’*lite* and *PAN* soybean oil.

SLF’s survey of households in Philippi also included questions about brand loyalty (SLF, 2018; Charman et al., 2019). Respondents were asked whether they thought brand or price was most important when purchasing maize and oil. 70% of respondents thought the brand was more important when buying maize, against 37% for cooking oil. When asked about their favourite brands, 88% of respondents said they preferred *White-star* flour. It is therefore clear that when instituting food policy, it is not merely financial aspects that need to be considered, but also other socio-cultural factors such as brand loyalty and perceived quality.

### 3.3 Hamper supply chain

Compared to other African countries, South Africa has a ‘highly developed and competitive formal retail market’, with both corporate and independent chains, representing in total 70% of sales, compared to 30% from the informal market (Trade Intelligence, 2016:62). The grocery retail sector is dominated by few actors; as shown in Table 4, Shoprite Holdings holds 26% market share, followed by Massmart-Walmart with 20%, Pick n Pay group (16%) and Spar Group (20%) (Trade Intelligence, 2016), all of which sell hampers.

**Figure 6:**
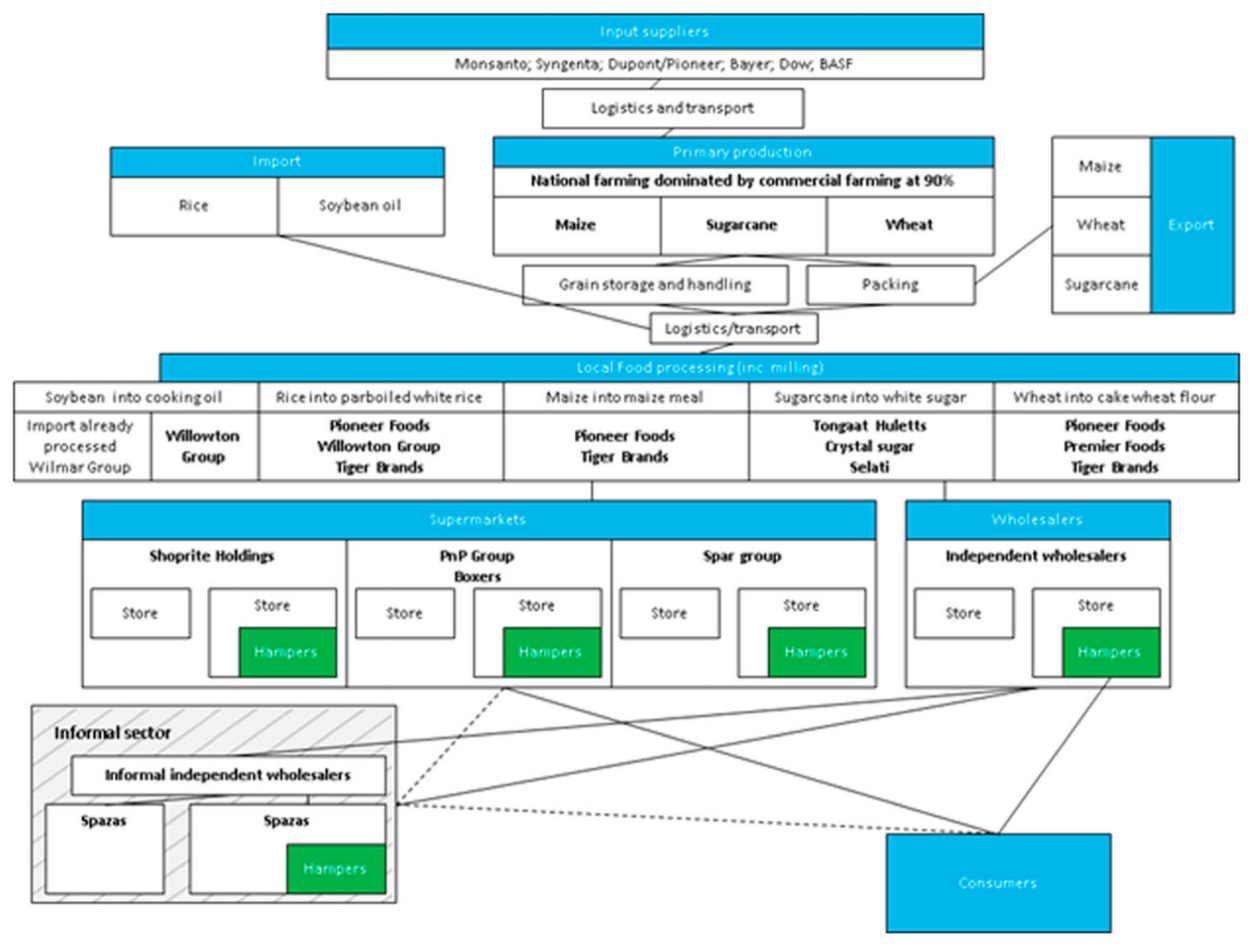
Hamper supply chain (made by authors, adapted from Greenberg, 2017).

The actor mapping (Figure 6) reinforces that the hamper supply chain is dominated by large, powerful actors, except for informal traders supplying their smaller share of hampers directly to consumers.

Most products included in hampers are produced by 6 companies: Pioneer Foods, Willowton Group, Premier Foods, Tiger Brands, Tongaat Hulett, and Wilmar (Figure 6). These food processors are South African, except for Wilmar Group headquartered in Singapore. Each of these companies sells several hamper staples; for example, Pioneer foods owns *Sasko flour, Spekko rice* and *Whitestar maize*. The sector is very concentrated, with the 5 top companies representing 20% of total income from the food processing and manufacturing industry in South Africa (Greenberg, 2015). The concentration ratio of the 5 largest companies (‘CR5’) is particularly high in the sugar industry (87%) where Tongaat Huletts, the 2nd largest sugar producer in the country and owns 4 of the 14 national sugar mills (DAFF, 2014a), held 22% of the market share in 2017 (Tongaat Huletts, 2017), as well as the grain and bakery industry, which includes wheat and maize production (CR5 of 75%) (Greenberg, 2015).

### 3.4 Nutritional content

The EAT-Lancet Commission’s healthy reference diet is composed of foods from 5 food groups (grains, vegetables, fruit, protein, and dairy) in addition to small amounts of added fats and sugars (Willett et al., 2019). Table 5 shows the nutritional information of a sample hamper found during fieldwork, based on the information provided by the products’ labels. The hamper was composed of *Whitestar* maize meal, *Sasko* flour, *Spekko* rice, *Crystal* sugar and *D*’*lite* cooking oil. Although the nutritional information may differ slightly between products brands, the table shows that, of the 5 recommended food groups, hampers only provide grains, in addition to sugar and fat. Although hampers in the formal sector are described by many interviewees as containing ‘all the necessities’, they do not include any major source of protein, fruit, vegetables, or dairy required for a healthy diet. These are purchased separately (either in the form of fruit and vegetable hampers) or on a more regular basis.

The EAT-Lancet’s healthy reference diet recommends that the proportion of energy intake from carbohydrates should be kept to less than 60% and constitute mainly of whole grains (Willett et al., 2019). Hampers contain high levels of refined carbohydrates in the form of cake wheat flour, white rice, and maize meal. Refining grains leads to a major loss of nutrients and fibre which in turn has important health implications, whilst whole grains and fibre have been associated with reduced risk of coronary heart disease, type 2 diabetes, and overall mortality (Willett et al., 2019).

Secondly, hampers bring large amounts of sugar: on top of a 10 kg bag of white sugar, cake wheat flour and maize meal both contain varying quantities of sugar. For reference, findings from the EAT-Lancet report suggest an intake of no more than 31g/day whilst the consumption of 10 kg of sugar by a household of 5, over a month would amount to over 66g/day of sugar per person.

Moreover, cooking oil included in hampers provides households with an important source of fat often added to starch-based meals to add flavour. In most hampers observed, the cooking oil was soybean oil.

Hampers are poor in protein, fibre, iron, and vitamins as they do not provide any of the 4 other food groups of the EAT-Lancet’s reference diet (Willett et al., 2019). Only maize meal provides any vitamins thanks to the national mandatory fortification programme on maize and bread flour which requires manufacturers to add iron, zinc, Vitamin A, thiamin, riboflavin, and Vitamin B (Labadarios et al., 2007). However, unlike bread flour, cake wheat flour which is consistently found in hampers is not fortified. Furthermore, it is clear that the fortification programme does not actually meet 100% of dietary needs according to Pretorius & Schönfeldt (2012). Their analysis of fortified maize meal found that Recommended Dietary Allowance for Vitamin A are not met for 1-9-year-olds, likely due to inadequate control systems and enforcement mechanisms over milling companies on which proper fortification depends.

Battersby (2011), Crush & Frayne (2011) and Charman et al. (2019) found evidence that people purchase food in bulk monthly from supermarkets upon reception of social grants, while daily and weekly shopping is done in informal shops to complement when and if their income permits it. This implies that a hamper may well represent the bulk of a households’ diet, although we do recognise that some level of supplementation with fresh produce and protein is likely. This is supported by the analysis of store manager interviews; **SM2** described hampers as containing a household’s ‘necessities’ that will hold them for a month and **WM3** said households will ‘make do during the month with whatever money they have left’. If a household is unable to afford to complement the hamper with additional sources of protein, fruit vegetables and dairy, it is evident that its diet is far from reaching the healthy reference diet recommended by the EAT-Lancet Commission (Willett et al., 2019). There is evidence to suggest that a healthy diet (let alone a sustainable diet) based on South Africa’s Food-Based Dietary Guidelines is indeed not within budget for most low-income South African households (Schönfeldt et al., 2013).

### 3.5 Are hamper contents substitutable?

When asked whether they saw potential to change the contents of the hampers, interviewees expressed that this would be difficult. The first reason was because of the brand loyalty consumers feel for certain products; for example, **WM1** said that they ‘stick to the same brands the customer wants’ and **WM2** explained that in their experience, changing brands leads to lower hamper sales; ‘we offer another brand […] but it’s not as popular as the top seller’. **WM3** said ‘I’ve tried with other brands, but it’s not as effective’. **SM4** explained that there was a ‘risk’ in offering a hamper without including the most popular brands, especially in the context of extremely high competition with neighbouring stores.

The interviews conducted with store managers show a lack of concern about the potential health impact of their hampers. When managers were asked if there is an opportunity to swap in healthier foods, such as wholegrains, interviewees dismissed the question and explained that this was not the point of hampers. Moreover, **SM4** explained that customers find them ‘healthy enough for them’ and that ‘their ancestors, parents and generations before them lived on the same thing and lived long healthy lives’ (**SM1**). Other answers included: ‘they don’t want healthier options’ (**WM2**). Store managers demonstrated a lack of knowledge about the importance of diverse and nutritious diets. However, **SM6** described fruit and vegetable hampers (Figure 4) as quite successful and popular among customers.

## 4 Discussion

This study first confirms the findings of Bowden et al. (2018) that hampers, also referred to as ‘combos’ are frequently purchased by low-income households in Cape Town and are widely available in stores across the city. The term ‘hamper’ designates a specific product; a bundle which consistently includes maize meal, cake wheat flour, vegetable oil, white sugar, and white rice, sold in bulk quantities. The empirical research goes further and finds that there is little diversity in the brands included in hampers, most products came from a few selected brands owned by 5 main processing companies.

### 4.1 Hampers and affordability

Hampers, and bulk-buying more generally, are purchased primarily by low-income households, as a strategy to access food cheaply (Battersby, 2011; Bowden et al., 2018). It is therefore a key area for interventions towards offering healthier food options to low-income families in conjunction with other policies aimed at improved food and nutrition security. The African Food Security Urban Network (AFSUN) measured that 66% of households in Cape Town purchase (some) food from informal markets or street food sellers, compared with 94% from supermarkets, from where people are most likely to purchase their bulk food (Battersby, 2011). This empirical study confirms that hampers are found in supermarkets, but also in wholesalers and in some spazas. Hampers were only found in the Cape Flats primarily in stores located close to, or within informal settlements such as in Philippi and Khayelitsha. Hampers were found in 5 stores across Khayelitsha and Philippi, townships which the AFSUN study found suffer from extremely high food insecurity rates (89% and 84% respectively) (Battersby, 2011). As the effects of the nutrition transition are also primarily felt among populations in these urban areas (Battersby, 2013), hampers ought to be further researched when looking at strategies to fight the effects of the multiple burdens of malnutrition.

In addition to the location of the stores, hampers were sold by stores which typically target low-income consumers (Shoprite, Boxers, Spar) as opposed to Pick n Pay, or Woolworths. The interviews conducted with store managers also support this finding. All interviewees working in stores which sold hampers described their customers as being very price-sensitive; customers were described as belonging to the LSM 1-4^1^ grouping (**SM1**), as heavily unemployed (**SM4**), or as ‘the bottom end, the poor, the needy’ (**WM3**). Although we can fairly affirm that food-insecure households are purchasing hampers, Bowden et al. (2018) found that households that do not have access to a monthly income or social grants are sometimes unable to afford this basic resource. Price constitutes the most important factor influencing a household’s decision of where to purchase hampers (Bowden et al., 2018). This empirical research finds that certain store managers use low-priced hampers as ‘draw cards’ to attract customers inside their stores (**SM1**, **SM4**), as they observe extremely high competition among retailers operating on low margins. Whilst hampers are generally sold at a discounted price compared to buying products individually, further price analysis found that this is not always the case (Table 3). This means that households are purchasing food that they perceive to be the cheapest, rather than on the actual price of the hampers. This offers an opportunity to include healthier options so that consumers have a legitimate choice as to what combination of dry goods they purchase.

### 4.2 Hampers and brand loyalty

Bowden et al. (2018) found that 3 additional factors influence households’ purchasing of hampers and groceries which are (1) location of the store (how far, how long to get there, how expensive to get there); (2) whether the store gives access to credit; and (3) quality of the food. This study does not provide additional evidence regarding these 3 factors but finds that brand loyalty plays an important role; the manager interviews, the survey data and the store-level data all show that brands included in hampers are carefully picked to meet customer demands. Over half of managers interviewed spoke of the importance of brand loyalty; particularly, *Whitestar* maize and *Spekko* rice were described as their customers’ favourites and were present in nearly all hampers. SLF found that 70% of respondents thought the brand was more important than price when buying maize, and that their favourite brand was *Whitestar*. The attraction customers feel to certain brands may be limiting the possibility of diversifying the contents of hampers, in a context of extremely high competition among retailers, who risk losing customers if they do not offer the preferred products. However, supermarket chains invest enormous sums in advertising to shape consumer spending. In 2018, Shoprite spent more than 1.4 billion rand on advertising, making it the country’s highest advertiser, all sectors included, with Pick n Pay and Massmart among the top 10 (Thompson, 2019). Brand loyalty and advertising should be leveraged to promote the sales of healthier and more sustainable hampers. The current industry self-regulatory approach adopted in South Africa is not delivering in terms of restricting advertising of unhealthy foods (Yamoah et al., 2021). Comprehensive public policies are therefore needed to strictly control the use of advertising techniques for unhealthy foods, in particular towards children.

### 4.3 Hampers and diets

The nutrition transition taking place in South Africa is characterised by a move towards poorly nutritious Westernised diets containing higher levels of fat, sugar, and processed foods (Popkin & Gordon-Larsen, 2004). Populations living in informal urban areas eat particularly poorly diversified diets (Labadarios et al., 2011). Bowden et al. (2018) found that hampers contain maize, rice, flour, cooking oil and sugar. This study goes further and found that more specifically, hampers generally contain cake wheat flour, white sugar, cooking oil, white parboiled rice, and maize meal, usually sold in packages of 10 kg and 2 l for cooking oil. They also suggest that ‘hampers are a blessing and a curse’ as their low price ensures families are fed, but may have serious long-term health implications due to their nutritional inadequacy if not well complemented with other more nutritious foods (Bowden et al., 2018:326). In fact, hampers contain nearly exclusively refined grains in the form of white cake wheat flour and white rice. These additional findings allow for a nutritional analysis of hampers.

Hampers contain energy-dense, nutrient-poor foods, high in carbohydrates, fat and sugar while lacking vitamins, iron, and fibre. Prioritising these foods in hampers that are used mainly by low-income households as key food security strategies will reinforce negative nutrition outcomes in these communities, but at the same time offer an opportunity to offer healthier options if they meet affordability and preference requirements.

If not complemented with sufficient fruit, vegetables, protein and dairy, a hamper-based diet cannot provide a healthy diet as defined by the EAT-Lancet commission (Willett et al., 2019). Hampers illustrate well the foods that are increasingly eaten in the nutrition transition described by Popkin et al. (2012). The low cost of starch-based staple foods often makes them the only affordable foods to many South African low-income households (Mchiza et al., 2015). Urban low-income households for whom hampers constitute an important portion of the food basket, may not be able to complement it with more nutritious foods providing the necessary protein, vitamins, and other micronutrients, and are likely to be eating energy-dense, nutrient-poor diets. Hampers can help provide calories to food insecure households, but a diversity of heal-thier products should be included to help promote healthier and more sustainable diets and address increasing multiple burdens of malnutrition. Making fruit and vegetable hampers a more prominent option – in conjunction with more traditional hamper – and including products such as lentils and tinned fish that can sometimes be found in hampers from spaza shops (see Figure 3) could be a first step in addressing the nutritional imbalance of hampers as a food security strategy.

Furthermore, there is a need for more coherent food policy to align with sustainable and healthy food systems. First introduced in 1991, the intention of the VAT-free policy exempting certain ‘basic foodstuffs’ was to make food more affordable for low-income households. At the time of the field study in July 2018, this list of ‘basic foodstuffs’ included 3 of the 5 hamper staples; cooking oil, maize meal and rice, whilst sugar and cake wheat flour remained taxed. In April 2019, in an attempt to compensate for the increased general VAT rate from 14 to 15% in 2018, white bread flour and cake wheat flour were added to the list of VAT-free items, by recommendation of an independent panel of experts to the Ministry of Finance (National Treasury, 2018). Although the report described the items as ‘refined carbohydrates and not necessarily the healthiest food option’, it recommended that they be made VAT-free due to their increased consumption by ‘the poor’ (National Treasury, 2018:47). This contradicts the emphasis of the Food and Nutrition Security strategy of 2014 that emphasises the need for more sustainable and healthy food systems that can address not only the scourge of hunger in South Africa, but also the growing problem of diet-related diseases and obesity. Whilst reducing rates of food insecurity should remain a priority for South Africa, this must be done by rendering healthy and nutritious food more affordable, instead of refined carbohydrates, the increased consumption of which is leading to other forms of malnutrition. In short, there needs to be better coherence between health and finance policies to tackle multiple burdens of malnutrition.

### 4.4 The potential of hampers as a strategy for more affordable, sustainable diets

As civil society and scientists are calling for deep changes to be made within food systems, locally and globally, to address the enormous sustainability and health challenges countries are facing, (Von Bormann, 2019; Willett et al., 2019), hampers could become a key leverage point to bring more diverse, healthy, and sustainable diets to urban, low-income households. This will require substantive concerted efforts to implement deep changes, including introducing locally produced nutrient-rich crops from sustainable agriculture. The key role of individual store managers in the assembling and selling of hampers, within a corporate-dominated supply chain is important to note as they may have the agency to start an initial alternative ‘sustainable and healthy’ hamper offering to customers. Incentives for them to do this can both be based on regulations from public policy, but also to be frontrunners in addressing food insecurity concerns. Having such a concentrated set of actors in the hamper supply chains means that it is only necessary to get a few companies to experiment with more sustainable and healthy alternatives in the hampers that they offer to see direct impacts on the ground.

There are indications that modifying hampers into healthier and more sustainable options is possible. Firstly, hampers comprising fruits and vegetables are increasingly sold in supermarkets targeting both higher and lower income customers, showing that bulk-buying culture is extending to fresh produce. Secondly, the interviews with store managers found that decisions regarding the sale and content of hampers lay with individual store managers. This highlights the need to work with retail store managers, to evaluate with them the possibilities and barriers to including healthier items in hampers and provide them with incentives to make these changes financially feasible and stimulate demand for healthy products. This should be done both with supermarkets and wholesalers who serve informal markets such as spazas where hampers were also found to be sold. This first implies challenging the retailers’ and consumers’ current understanding of what ‘the basic necessities’ are. Finally, the brand loyalty consumers feel towards certain products could and should be leveraged to encourage healthier eating by encouraging food processing companies to develop healthier alternatives. These could include whole grains instead of refined grains, and a wider variety of foods such as pulses, legumes, nuts, and fruit as recommended by the EAT-lancet Commission (Willett et al., 2019). VAT-free ‘basic foodstuffs’ include a number of foods such as dried beans, lentils, sardines, dairy, vegetables, fruit, eggs, and legumes, a range much closer to the one recommended by the Eat-Lancet Commission (2019). Aligning retailers’ and food processors’ understanding of ‘basic necessities’ to the one provided by the South African government would be a first step towards improving the nutritional quality of hampers.

To reduce the environmental footprint of hampers, priority should be put on identifying crops that can be produced under diversified agroecological systems to replace those reliant on monoculture production in order to build long-term fertility and healthy agro-ecosystems (IPES-Food, 2016). Alternatives to current hamper staples could thus include nutrient-rich plants such as legumes, which can directly contribute to improving soil quality when used as cover crops, and contain soil erosion. Certain plants native to South Africa such as sorghum, sweet potato, and cowpea are also better adapted to local climates, and their production would contribute to protecting the country’s agricultural biodiversity (Biodiversity International, 2017). Environmentally sustainable forms of agriculture must continue to be promoted; to this end, alternative agricultural solutions such as agroecology should be institutionalised, requiring increased public investment into agroecological research and enabling policy environments (Pereira et al., 2018).

In order to bring about changes to the contents of hampers, government subsidised programmes targeted at retailers could prove helpful. One useful example comes from the Scottish Grocers Federation’s Healthy Living Programme running since 2004, which aims to improve the supply and provision of fresh produce and healthier food in convenience stores, particularly those in more deprived areas. The programme, subsidised by the Scottish government, supports retailers to promote healthier diets, by delivering trainings and resources to help them maximise the sales of healthy food choices and minimise waste (Leven, 2013). Such a programme could be designed to support a number of Cape Town retailers to experiment with improving the content of hampers. For example, the programme could aim to educate participating retail managers about healthy and sustainable food options, help them to identify alternatives to include in hampers, provide promotional and educational material directed as customers to be used in stores so as to boost sales of healthy and sustainable hampers, help reduce the environmental impact of food waste by including perishables close to their sell-by date in hampers, and provide monetary support to cover financial risks.

In designing such programmes and store-based interventions, it is important to consider the cultural component of diets and pay particular attention to women’s needs and shopping habits as they are found to be making most food-related decisions, including purchasing of hampers (Bowden et al., 2018). Although government subsidised programmes may be costly, the potential of such interventions could be great by supporting retailers, including financially, to experiment with healthier and more sustainable hampers.

## 5 Conclusions

In this paper, we set out to explore the potential role that hampers could play in achieving more sustainable diets in South Africa. Based on empirical data collected in Cape Town, hampers were confirmed as important strategies for low-income households to meet caloric requirements and fulfil satiation needs. The content of hampers was shown to include the following 5 staples: cake wheat flour, white sugar, parboiled white rice, soybean cooking oil and maize meal. The nutritional aspect of hampers as they are currently configured, were analysed and found to be significantly lacking from recommendations made by the EAT-Lancet Commission (Willett et al., 2019). There is thus room for improvement by leveraging the power of hampers as a strategy for low-income households and looking for alternative products that better contribute to sustainable diets. However, given the existence of fruit and vegetable hampers in some instances – as well as the inclusion of some more nutritious foods like lentils and tinned fish in some hampers, there is evidence of potential for this food security strategy to be leveraged to promote sustainable diets and make them accessible to low-income households facing high rates of food insecurity and malnutrition.

Further research is needed to develop knowledge in this area. In particular, a comparison of the consumption of hamper staples with other foods, as well as a deep evaluation of hamper consumers’ diets, to understand whether hampers are being adequately complemented with other nutritious foods, or how they can be. An evaluation of possible substitute foods for hamper staples, which would provide better nutrition, have a lower environmental impact, and yet which are susceptible to provide an attractive hamper to low-income urban households would be a key next step. Further research could also help identify the possibilities for cooperation with food retailers and wholesalers serving informal spaza shops, in order to incentivise them to leverage their power and essential role in improving consumers’ diets. The role of public policy in creating an enabling environment for – and incentivising – such interventions is another important area for further research. By paying careful attention to variables including price, convenience, culture and brand loyalty, we can hope not only to improve the access of households to nutritious food, but to improve their access to high-quality sustainable foods.

## Figures and Tables

**Table 1:** Research questions and methods used.

Research Questions	Method used
What is a hamper and which foods does it typically include?	Collection and analysis of store-level data
	Qualitative interviews with store managers and thematic analysis
Who purchases hampers, and how?	Qualitative interviews with store managers and thematic analysis
	Quantitative analysis of SLF survey data
What are the nutritional and environmental implications of hampers?	Nutritional analysis based on analysis of store-level data collected and literature
	Environmental analysis based on analysis of store-level data collected and literature
What is the origin of hampers and which actors are most involved in their supply chain?	Qualitative interviews with store managers and thematic analysis
	Collection and analysis of store-level data
	Mapping of supply chain actors based on qualitative interviews and literature
What are the barriers and opportunities to improving the contribution of hampers to sustainable diets?	Qualitative interviews with store managers and thematic analysis
	Mapping of Value Chain actors based qualitative interviews and literature

**Table 2:** Stores where interviews were conducted.

Store type	Interviewee	Area	Store sold hampers
Supermarket	SM1^a^	Mitchell’s plain	Yes
Supermarket	SM2	Constantia	No
Supermarket	SM3	Philippi	Yes
Supermarket	SM4	Philippi	Yes
Supermarket	SM5	Khayelitsha	Yes
Supermarket	SM6	Bellville	No
Supermarket	SM7	Philippi	No
Wholesaler	WM1^b^	Philippi	Yes
Wholesaler	WM2	Philippi	Yes
Wholesaler	WM3	Epping	Yes

a SM stands for ‘Supermarket Manager’.

b WM stands for ‘Wholesaler Manager’.

**Table 3:** Content and price of hampers found in Cape Town, and estimated savings.

Hamper number	Hamper content	Quantity		Estimated price if sold individually	Actual hamper price	Estimated savings	% savings
1	rice	10 kg		R356.50	R349.00	R7.50	2%
	sugar	10 kg					
	oil	2 l	*				
	flour	10 kg					
	maize	10 kg					
2	rice	10 kg		R369.50	R369.00	R0.50	0%
	sugar	10 kg					
	oil	2 l	*				
	flour	10 kg					
	maize	10 kg					
3	rice	10 kg		R404.34	R385.00	R19.34	5%
	sugar	10 kg	*				
	oil	2 l	*				
	flour	10 kg					
	maize	10 kg					
4	rice	10 kg		R417.34	R395.00	R22.34	5%
	sugar	10 kg	*				
	oil	2 l	*				
	flour	10 kg					
	maize	10 kg					
5	rice	10 kg		R384.50	R369.00	R15.50	4%
	sugar	10 kg					
	oil	2 l	*				
	flour	10 kg					
	maize	10 kg					
6	rice	10 kg		R371.50	R349.00	R22.50	6%
	sugar	10 kg					
	oil	2 l	*				
	flour	10 kg					
	maize	10 kg					
7	rice	10 kg		R373.50	R375.00	−R1.50	0%
	sugar	10 kg					
	oil	2 l	*				
	flour	10 kg					
	maize	10 kg					
8	rice	10 kg		R401.91	R494.95	−R93.04	−23%
	sugar	10 kg					
	oil	2 l					
	flour	10 kg					
	maize	10 kg					
9	flour	10 kg		R251.93	R239.99	R11.94	5%
	rice	10 kg					
	maize	10 kg					
10	flour	10 kg		R221.97	R345.00	−R123.03	−55%
	rice	10 kg					
	maize	10 kg					
11	rice	10 kg		R359.96	R360.00	−R0.04	0%
	sugar	10 kg					
	oil	2 l					
	maize	10 kg					
12	rice	5 kg		R235.45	R229.95	R5.50	2%
	sugar	5 kg					
	oil	2 l	*				
	flour	5 kg	*				
	maize	5 kg					
13	rice	10 kg	*	R280.15	R229.00	R51.15	18%
	Extra porridge	5 kg					
	flour	10 kg					
	maize	10 kg					
14	flour	10 kg	*	R265.82	R239.99	R25.83	10%
	rice	10 kg	*				
	maize	10 kg					

* Products for which the price when sold individually was not available. Therefore, the price is estimated as the average price at which that product is sold across all stores visited.

**Table 4:** Major South African grocery retail groups, according to market share (Trade Intelligence, 2016).

Grocery retail group	Include	National Market Share
Shoprite Holding	Shoprite	26%
	Checkers	
	U-Save	
Massmart Holdings	Makro	20%
	Game	
	Jumbo Cash & Carry	
Spar Group	Spar	20%
	SuperSpar	
Pick n Pay	Pick n Pay	16%
	Boxer	
Woolworths		<15%

**Table 5:** Macro and micronutrient content of sample hamper observed during fieldwork, based on nutrition labels.

	Maize meal (100 g)	Cake wheat flour (100 g)	Parboiled white rice (120 g cooked)	White sugar (100 g)	Cooking oil (100 ml)
Food Group	Grains	Grains	Grains	Sugar	Fat
Energy (kj)	1368	1439	659	1692	3389
Protein (g)	5.6	10.2	3.2	0	0
Glycaemic carbohydrates (g)	72	71	34	100	0
of which total sugars (g)	1.8	0.2	1.4	99.8	0
Total fat (g)	0.7	0.9	0.4	0	91.7
of which saturated fat (g)	0.1	0	0.1	0	14
of which trans fat (g)	<0.01	<0.1	<0.05		<0.5
of which	0.2	0	0.1		26
monounsaturated fat (g) of which polyunsaturated fat (g)	0.4	0	0.1		55
cholesterol (mg)	<1	<1	<1		0
Dietary fibre (g)	2.5	3.7	1.3		
Total sodium (mg)	<6	<5	<6	<1	0
Vitamin A (μgRE)	188				
Thiamine (B1) (mg)	0.3				
Riboflavin (B2)	0.2				
Niacin (B3) (mg)	3				
Pyridoxine (B6)	0.4				
Folic acid (B9) (μg)	189				
Iron (mg)	3.7				
Zinc (mg)	1.9				

## Footnotes

1 The Living Standards Measure or LSM is a marketing and research tool (similar to ‘social economic class’) used in South Africa to segment the population from 1 to 10 according to standard of living and disposable income (SAARF, 2017).
